# VacStent as a novel therapeutic approach for esophageal perforations and anastomotic leaks– a systematic review of the literature

**DOI:** 10.1186/s12893-025-03067-5

**Published:** 2025-07-21

**Authors:** Dimitrios Kehagias, Sameh Abogabal, Charalampos Lampropoulos, Muhammad Ijlal Haider, Ioannis Kehagias, Prashant Jain, Vincent Wong

**Affiliations:** 1 Department of Upper Gastrointestinal Surgery, Hull University Teaching Hospitals NHS Trust, Hull, UK; 2 Department of Surgery, University General Hospital of Patras, Patras, Greece; 3 Intensive Care Unit, Saint Andrew’s General Hospital of Patras, Patras, Greece

**Keywords:** Esophageal perforation, Anastomotic leak, Endoscopic vacuum therapy, VacStent, Upper Gastrointestinal tract

## Abstract

**Background:**

Perforation and anastomotic leak (AL) of the esophagus remain a dreaded complication for surgeons, endoscopists and patients. The VacStent is a novel endoscopic device, combining the benefits of a self-expandable metallic stent (SEMS) and endoscopic vacuum therapy (EVT). This systematic review aims to investigate the efficacy and clinical outcomes associated with the use of the VacStent.

**Methods:**

A systematic literature review was conducted in PubMed and Google Scholar electronic databases using keywords and Medical Subject Headings terms. Peer-reviewed studies in English language that reported the use of VacStent for transmural defects of any cause of the esophagus and gastroesophageal junction (GEJ) were included. Data regarding technical details of the VacStent, outcomes, and complications were extracted and presented in a narrative synthesis. Clinical success was defined as closure of the defect without requiring further intervention.

**Results:**

Of the 153 articles identified after searching, nine studies were deemed eligible and included in the final analysis. The methodological quality of the included studies was generally moderate. Sixty-five patients underwent VacStent treatment for the following indications: AL (70%), Boerhaave syndrome (10%), iatrogenic perforation (10%), and other causes (10%). The mean interval between VacStent changes was 5.3 (2–8) days. Prior endoscopic interventions, including SEMS, clips, and EVT, had been performed in 25 patients (38%). The mean duration of VacStent therapy was 8.8 ± 8.3 days. On average, each patient received between one and three VacStents. Technical success was achieved in all 65 cases (100%), while clinical success was observed in 50 patients (77%). Approximately 70% of patients were able to tolerate a liquid diet during treatment, and no major complications were reported.

**Conclusions:**

The VacStent appears as a promising tool, integrating the benefits of both EVT and SEMS, while allowing oral intake. The included studies present preliminary data, warranting cautious interpretation. Further evidence is required to delineate the clinical utility and appropriate indications for VacStent therapy.

**Trial Registration:**

PROSPERO database (UIN: CRD420251012718).

## Background

Esophageal or gastroesophageal junction (GEJ) defects, resulting from either perforation or anastomotic leak (AL), represent a severe and worrisome complication for endoscopists, surgeons and patients alike [1]. According to the literature, approximately 80% of esophageal perforations are iatrogenic, while spontaneous perforation, namely Boerhaave’s syndrome, accounts for about 15% of all cases [2, 3]. Postoperative AL following open or minimally invasive esophagectomy, remains a significant concern, with reported rates of up to 20% [4]. Optimal outcomes hinge on early recognition and prompt intervention in specialized tertiary care centers.

With the advent of latest technological advancements in endoscopy and improved technical skillsets among interventional endoscopists, more advanced procedures for malignant or benign lesions of the esophagus are performed. However, these complex interventions entail a higher risk of perforation. For instance, endoscopic submucosal dissection, stricture dilation, foreign body removal, endoscopic mucosal resection, tumor ablation or peroral endoscopic myotomy, comprise endoscopic therapeutic procedures with greater risk of perforation [5]. For surgeons, the risk of AL continues to be a significant complication following esophagectomy, despite ongoing efforts to mitigate this risk through technical refinements and the centralization of care in high-volume centers. Specifically, after esophageal resections, AL occurs in 10–20% of cervical anastomoses, and 5–10% of thoracic anastomoses [6]. Additionally, the rising prevalence of metabolic bariatric surgery, including mainly sleeve gastrectomy and Roux-en-Y gastric bypass, has further contributed to a higher incidence in postoperative AL [7].

Regardless of the underlying cause, a defect in the gastrointestinal (GI) tract allows the spill of luminal content into a sterile environment, triggering a systematic inflammatory response. This can rapidly progress to severe sepsis and in some cases can lead to mortality, while long-term complications such as fistula formation might occur. Mortality of esophageal perforation can be up to 50%, if initiation of treatment is delayed beyond 24 h [8]. Immediate priorities should include prompt resuscitation and hemodynamic stabilization of the patient. While surgical intervention was historically recommended as first-line therapy, recent evidence supports a stepwise approach of non-invasive endoscopic and interventional radiology measures, with surgical interventions reserved for those who do not respond to conservative management [9–11].

Advances in endoscopic techniques and biomedical device development have significantly expanded the available therapeutic options for endoscopists. These advancements allow managing defects of the GI tract in a minimally invasive way, thereby reducing even more the need for surgical intervention [12]. Employing endoscopic drain placement, in cases where drainage through chest or abdomen is not feasible, through the scope or over the scope clips, and the deployment of fully covered self-expanding metallic stent (SEMS) are only some of these advanced endoscopic techniques that facilitate healing and defect closure [13, 14].

One of the most important recent advancements for managing GI defects is the introduction of endoscopic vacuum therapy (EVT) devices, such as the EsoSponge^®^ (B. Braun, Melsungen, Germany). Emerging data suggests that EVT may be more effective than SEMS, as it combines continuous drainage with stimulation of anastomotic healing [15, 16]. Some studies further support that combining EVT with SEMS may enhance clinical outcomes. According to the latest guidelines from the American and European societies of gastrointestinal endoscopy, EVT is now considered a viable option for managing defects of the GI tract [17, 18]. Particularly in cases with esophageal perforation without rupture of the parietal pleura, EVT is recommended as a first line treatment [11]. However, it is crucial to recognize that SEMS or EVT might not be sufficient in cases with extensive intrathoracic contamination, and large cavities requiring additional drainage [12].

Given the limitations associated with both EVT devices and SEMS, growing evidence supports the use of the VacStent^®^ (Möller Medical GmbH, Fulda, Germany) as an emerging therapeutic alternative. The VacStent integrates a covered SEMS within a polyurethane sponge cylinder, combining the mechanical sealing capability of a stent with the benefits of vacuum therapy. This device promotes healing through continuous suction, debridement, and localized wound irrigation. This irrigation results in enhanced perfusion, increased oxygen delivery and formation of granulation tissue [19]. Preliminary clinical studies suggest that the VacStent may offer promising outcomes, with potentially fewer device exchanges compared to conventional EVT systems. Additionally, it maintains patency of the GI tract, enabling oral intake during treatment and potentially improving the overall quality of the hospital stay [20, 21].

Despite its potential therapeutic benefits, the current literature on VacStent use remains limited to preliminary data. There is a lack of prospective, comparative studies evaluating its efficacy against other established endoscopic techniques. Consequently, the precise role of the VacStent in the management of esophageal perforations or AL has yet to be clearly defined. The aim of this systematic review is to evaluate the efficacy and clinical outcomes associated with VacStent use in the treatment of esophageal and GEJ perforations or AL.

## Methods

### Search strategy

A systematic review of the literature was conducted, in accordance with the Preferred Reporting Items for Systematic Reviews and Meta-Analyses (PRISMA) guidelines [22]. The electronic databases Google Scholar^®^ and PubMed^®^ (National Library of Medicine, Bethesda, MD, USA) were searched for articles published between January 2019 and March 2025, since the VacStent became commercially available only from 2019 onward. The systematic review was registered in the PROSPERO database (UIN: CRD420251012718).

The search strategy utilized a combination of keywords and Medical Subject Headings (MeSH) terms, with Boolean operators. The specific search syntax included: “VacStent” AND “perforation” OR “esophageal leak” OR “leak” OR “leakage” OR “Boerhaave”. The advanced search syntaxis can be shown in the protocol registered in PROSPERO database. Three authors independently performed the literature search across both databases. Duplicate articles were removed prior to screening. The subsequent screening process involved reviewing titles and abstracts to exclude irrelevant studies. Additionally, the references of the retrieved articles were also examined to identify additional studies, not captured in the initial search. A fourth author served as a supervisor and resolved any discrepancies or disagreements regarding study selection.

The retrieved articles were rigorously evaluated for eligibility. Studies were included if they reported outcomes of VacStent use for perforations and AL of the upper GI tract of any cause. Inclusion criteria were as follows: (1) articles published in English (2), cohort studies—retrospective or prospective—and (3) case report studies. The exclusion criteria were: (1) editorials, commentaries, or conference abstracts (2), non-English language publications (3), books or book chapters (4), systematic or narrative reviews, and (5) studies focusing on EVT without specific reference to VacStent. Additionally, studies involving duplicate patient cohorts were excluded.

### Data extracted and analysis

For each included study, the following data were extracted: author, year of publication, study design, number of patients and demographic characteristics, specifically age and gender. The etiology of the defect was recorded, along with detailed characteristics, such as the size of the defect and the associated cavity. Moreover, when available, the precise location of the defect was documented as the measured distance from the teeth.

Technical aspects of the VacStent procedure were also extracted, including details on insertion and removal techniques. The brand of the stent used, type of sedation administered, and criteria for VacStent placement were documented. Particular attention was paid to the interval between VacStent changes. Information regarding vacuum pump function—specifically suction pressure and the time for closure prior to VacStent removal—was also recorded. Additionally, the volume of saline injected before stent removal was evaluated and extracted.

Regarding the VacStent treatment several parameters were assessed across the studies. These included prior treatments attempted before VacStent placement, the total duration of VacStent therapy, and the average number of stents used per patient. To evaluate treatment efficacy, both technical and clinical success rates were extracted from each study. Technical success was defined as the successful application of the VacStent in the proper place without adverse events. Clinical success was defined as successful closure of the leak or perforation, without the need of further post-VacStent treatment.

Finally, outcomes and complications associated with VacStent use were thoroughly reviewed. Patient ability to resume oral intake, either liquids or solids, and the need for further interventions post-VacStent therapy were evaluated. Reported complications included sponge dislodgement, narrowing of the esophageal passage, bleeding, and stent migration.

Continuous variables are expressed as mean ± standard deviation (SD); categorical variables as counts (percentages). Given the substantial clinical and methodological heterogeneity among the nine included studies—including differences in design, intervention protocols, outcome definitions, and reporting completeness—a formal quantitative synthesis (meta-analysis) or subgroup analyses (e.g., by etiology or prior endoscopic intervention) was not feasible. We therefore present a structured narrative synthesis to summarize the available evidence. To evaluate the potential confounding effect of prior endoscopic interventions, patients were stratified into treatment-naïve and previously treated groups, and leak-closure rates were compared using Fisher’s exact test.

### VacStent GI™

The VacStent GI™ is a medical device developed by VAC Stent GmbH and distributed by MICRO-TECH Europe GmbH [23]. It comprises a delivery system preloaded with a SEMS encased in open-pore polyurethane foam. The SEMS is made of nitinol wire and is internally coated with a silicone layer to prevent tissue ingrowth. The external surface of the stent is enveloped in open-pore polyurethane foam, which is connected to a blue drainage tube.

**Fig. 1:**
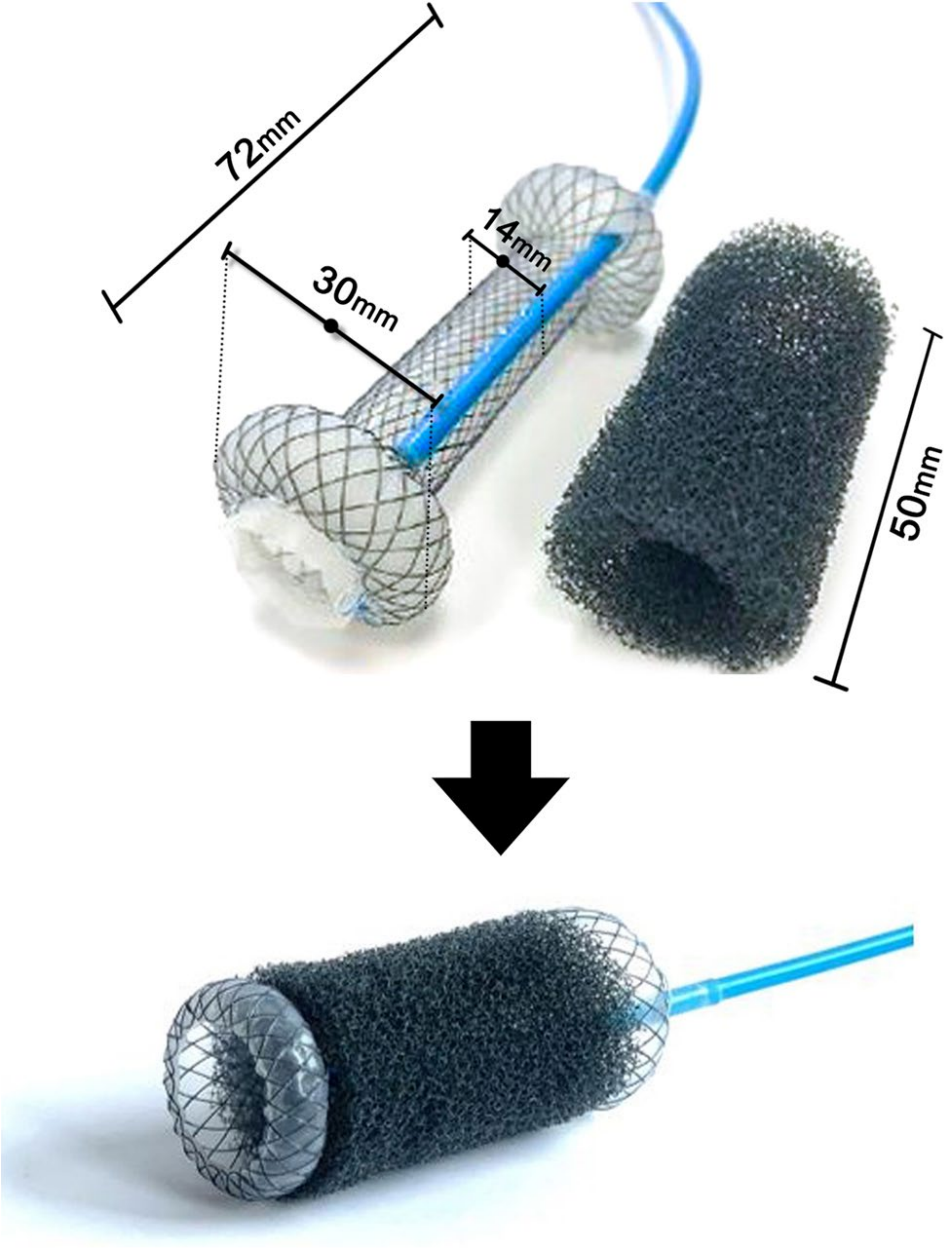
VacStent device

When fully expanded, the VacStent forms a dumbbell shape, with a central body diameter of 14 mm and flared ends of 30 mm, a design that aids in preventing migration (Fig. 1). The device is currently available in a single size, with a length of 720 mm and a diameter of 14 mm.

The VacStent integrates two established therapeutic approaches for managing anastomotic leaks or gastrointestinal perforations. The SEMS component seals the defect, while the sponge facilitates continuous suction and drainage of the wound cavity, promoting granulation tissue formation. A significant advantage is the preservation of gastrointestinal continuity, allowing oral intake during treatment. The device is indicated for defects measuring up to 3 cm in size.

### Quality assessment

Two authors independently assessed the quality and risk of bias of the studies that met the eligibility criteria and were included in the review. Case reports were evaluated using the Joanna Briggs Institute (JBI) Critical Appraisal Checklist for Case Reports; case series (studies with more than 1 but fewer than 10 patients) were assessed using the JBI Critical Appraisal Checklist for Case Series; and all other studies were evaluated using the Methodological Index for Non-Randomized Studies (MINORS) tool [24, 25]. A third author participated in the discussion and resolution of any discrepancies, while a senior author supervised the entire process. Based on the score of the MINORS tool, a score of 8 was defined as poor quality, 9–14 as moderate quality, and 15–16 as good quality.

## Results

### Screening process and study characteristics

**Fig. 2:**
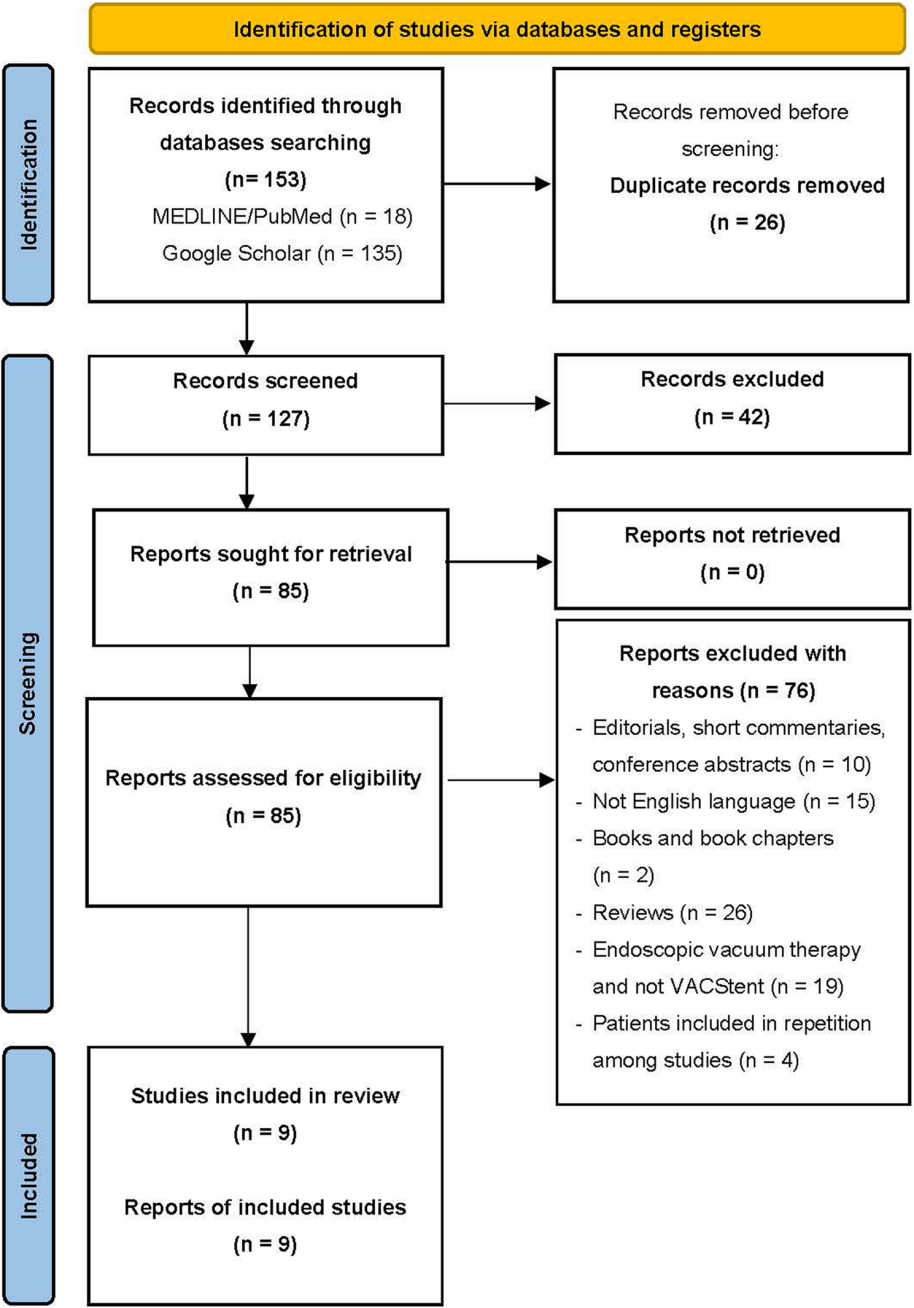
PRISMA Flowchart

**Fig. 3:**
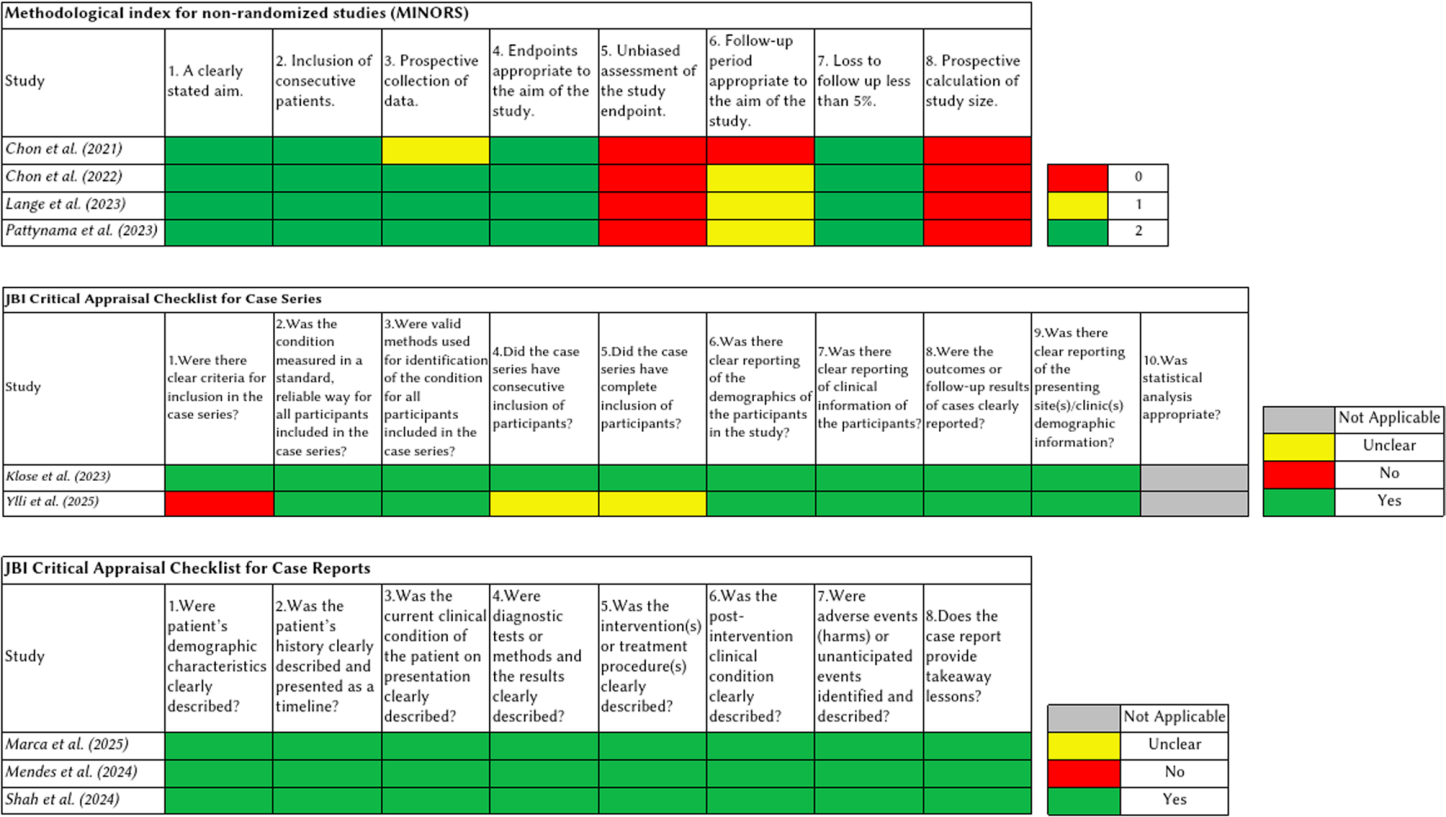
Assessment of methodological quality

Figure 2 illustrates the systematic process used to identify and select studies for inclusion. Following the initial identification of the articles, duplicates were removed, and a preliminary screening was performed based on titles and abstracts, excluding any irrelevant articles. Then, the inclusion criteria were applied for assessing the eligibility of the remaining articles. After the review process, nine studies were deemed eligible and were included in the final analysis (Table 1) [26–34]. The results of the quality assessment are summarized in Fig. 3.

**Table 1:** Characteristics of included studies

Author	Year	Type of study	Number of patients (*n*)	Age (y)	Gender	Etiology of defect (*n*)	Defect characteristics		
							Distance from the teeth (cm)	Defect size (cm)	Cavity size (cm)
Chon et al. [26]	2021	Retrospective single-center	10	60.1 ± 17.6 ^*^	M (8/10) F (2/10)	AL (7) BS (2) Iatrogenic (1)	34.7 ± 5.9 ^*^	1.4 ± 0.8^*^	2.8 ± 0.6^*^
Chon et al. [27]	2022	Prospective single-center	20	61.3 ± 11.8	M (20/20)	AL (18) Iatrogenic (2)	30.75 ± 7.5	1.1 ± 0.7	2.1 ± 2
Lange et al. [28]	2023	Prospective multi-center	15	70.5 ± 10.9	M (10/15) F (5/15)	AL (9) Iatrogenic (3) Hiatal (1) SG (1) LINX (1)	27.9 ± 5.4	1.5 ± 0.88	3.6 ± 2.9
Klose et al. [29]	2023	Case series	3	55.6 ± 14	M (2/3) F (1/3)	AL (2) BS (1)	.	.	.
Pattynama et al. [30]	2023	Prospective single-center	10	66.6 ± 9.4	M (10/10)	AL (8) BS (1) Iatrogenic (1)	.	.	.
Marca et al. [31]	2024	Case report	1	30	F	SG	.	.	.
Mendes et al. [32]	2024	Case report	1	45	M	BS	.	.	.
Shah et al. [33]	2024	Case report	1	56	F	RYGBP	.	.	.
Ylli et al. [34]	2025	Case series	4	64.7 ± 14.8	M (3/4) F (1/4)	AL (2) BS (2)	.	.	.

AL: anastomotic leakage; BS: Boerhaave syndrome; SG: Sleeve Gastrectomy; RYGBP: Roux-en-Y gastric bypass

*Expressed as mean ± standard deviation

All included studies were published between 2021 and 2025, following the commercial availability of the VacStent in 2019. The studies comprised three single-center cohort studies—one retrospective and two prospective—and one prospective multicenter study. The remaining five publications were case report studies and case series. In total, 65 patients underwent VacStent treatment, of whom 54 were male and 11 females. The mean age of patients across studies ranged approximately from 60 to 70 years.

Regarding the etiology of the defect, AL was the most frequently reported, accounting for 46 cases (70%). Boerhaave syndrome and iatrogenic perforations each accounted for seven cases. Additionally, three cases involved post-bariatric surgery leaks—two following sleeve gastrectomy and one after Roux-en-Y gastric bypass. Two atypical cases were also reported: one following hiatal hernia repair and another due to erosion at the esophagogastric junction caused by a LINX^®^ (Johnson & Johnson, New Brunswick, NJ, USA) device. The characteristics of the leak size and location were demonstrated in only three of the included studies as shown in Table 1. Across the three studies, the mean defect size was 1.3 ± 0.78 cm, and the mean cavity size was 2.75 ± 2.20 cm. Regarding the exact location of the defects, the mean distance from teeth was 30.6 ± 6.8 cm.

### Technical details of VacStent

All studies utilized the VacStent GI™, which is currently the only commercially available patented device. In studies that specified the type of anesthesia, VacStent placement was performed either under deep sedation with propofol or under general anesthesia, indicating its feasibility in both settings. The primary criterion for VacStent placement was the ability to fully cover the defect, typically limited to defects less than 3 cm in length.

There was considerable variability among the studies regarding the recommended time interval for VacStent change, as summarized in Table 2. Based on available data, the average interval for changing the VacStent was 5.3 days. Similarly, substantial variation was observed in the suction pressure applied by the vacuum pump, which ranged from 60 to 125 mmHg. In some studies, a different technique was described, employing a higher initial suction pressure on the first day, followed by a reduction in the subsequent days. Prior to VacStent removal, suction from the vacuum pump was discontinued at least two hours in advance, and 20 to 40 mL of normal saline was instilled to moisten the sponge. In some studies, saline injection (20 or 40 mL) was performed three times daily as part of routine management, not solely before the removal of the VacStent (Table 2).

**Table 2:** Technical details of vacstent insertion and change

Author	Stent manufacturer	Type of sedation	Criteria for stent position	Pressure suction of the vacuum pump (mmHg)	Days after each change (*n*)	Time of closure of the pump before change (hours)	Saline injected before removal (ml)
Chon et al. [26]	VacStent GI™	DS GA	Distance 1 cm from stent ends	125	2–4	2	20
Chon et al. [27]	VacStent GI™	DS GA	Distance 1 cm from stent ends	65	3–5	2	20
Lange et al. [28]	VacStent GI™	DS GA	Able to cover completely the defect	85 (75–125)	2–5	2–4	40
Klose et al. [29]	VacStent GI™	.	.	60–80	6–8	.	.
Pattynama et al. [30]	VacStent GI™	DS GA	Defects less than 3 cm	125 75 after day 1	5–7	.	20 TID
Marca et al. [31]	VacStent GI™	.	.	.	4–5	.	.
Mendes et al. [32]	VacStent GI™	.	.	120 80 after day 1	7	.	40 TID
Shah et al. [33]	VacStent GI™	.	.	125 75 after day 1	7	.	.
Ylli et al. [34]	VacStent GI™	GA	.	.	6	.	.

*DS *deep sedation; *GA* general anesthesia; *TID* three times a day

### VacStent treatment

Of the 65 patients who underwent VacStent treatment, 25 had received prior endoscopic interventions. Specifically, 18 patients were treated with EVT using the EsoSponge, four patients received a covered self-expandable metallic stent (SEMS), two patients had an over-the-scope clip placed, and one patient underwent both EVT and SEMS prior to VacStent placement. The total duration of VacStent therapy ranged from approximately 5 to 14 days. The mean number of VacStents used per patient ranged from 1 to 3, as detailed in Table 3.

**Table 3:** VacStent treatment overall and stratified according to prior endoscopic interventions

Author	Treatment before VacStent	Total duration of VacStent (days)	Mean VacStent per patient	Technical success (*n*, %)	Clinical success (all patients, *n* = 65) (*n*, %)	Clinical success (treatment-naïve cohort, *n* = 39) (*n*, %)	Clinical success (previously treated cohort, *n* = 26) (*n*, %)
Chon et al. [26]	EVT (2/10) OTSC (2/10) SEMS (1/10)	6.3 ± 4 ^*^	1.5/1	(10/10) 100%	(7/10) 70%	(4/5) 60%	(3/5) 80%
Chon et al. [27]	EVT (3/20)	4.8 ± 2.1	1.2/1	(20/20) 100%	(12/20) 60%	(12/17) 71%	(0/3) 0%
Lange et al. [28]	EVT (7/15)	14.6 ± 14	2.7/1	(15/15) 100%	(**12**/15) 83%	(6/8) 75%	(6/7) 85%
Klose et al. [29]	EVT and SEMS (1/3)	14 ± 12	3/1	(3/3) 100%	(3/3) 100%	(2/2) 100%	(1/1) 100%
Pattynama et al. [30]	EVT (6/10)	9.7 ± 4.5	1.4/1	(10/10) 100%	(**10**/10) 100%	(3/3) 100%	(7/7) 100%
Marca et al. [31]	SEMS (1/1)	13	3/1	(1/1) 100%	(1/1) 100%	-	(1/1) 100%
Mendes et al. [32]	SEMS (1/1)	7	1/1	(1/1) 100%	(1/1) 100%	-	(1/1) 100%
Shah et al. [33]	SEMS (1/1)	7	1/1	(1/1) 100%	(1/1) 100%	-	(1/1) 100%
Ylli et al. [34]	No treatment (0/4)	7.5 ± 2.5	1.25/1	(4/4) 100%	(3/4) 75%	(3/4) 100%	-

*EVT* endoscopic vacuum therapy; *OTSC* over the scope clip; *SEMS* self-expandable metallic stent

*Expressed as mean ± standard deviation (mean ± SD)

In the nine studies reviewed, “clinical success” was consistently defined as complete closure of the gastrointestinal leak without the requirement for additional endoscopic or surgical intervention. In two investigations, criteria were expanded to include restoration of oral intake and hospital discharge in a state of clinical recovery. Closure of the defect was universally confirmed by direct endoscopic inspection—either at the time of stent removal or during scheduled follow-up—demonstrating intact mucosal healing and absence of dehiscence. In seven studies, this endoscopic assessment was complemented by contrast-enhanced imaging (contrast esophagogram or CT with oral contrast) to rule out persistent extravasation.

Technical success was achieved in all 65 cases, with successful placement of the VacStent and no reported procedural adverse events. However, clinical success was reported in 77% (50/65) of patients, indicating that a subset required further therapeutic intervention following VacStent therapy. To determine whether prior endoscopic treatments influenced VacStent performance, we conducted a subgroup (sensitivity) analysis stratifying patients into treatment-naïve (*n* = 39) and previously treated (*n* = 26) cohorts. Both groups demonstrated identical clinical success rates of 77% (30/39 versus 20/26; *p* = 1.0 by Fisher’s exact test) (Table 3).

### Outcomes and complications of VacStent

Complications associated with VacStent therapy were relatively limited. Sponge dislodgement occurred in four patients, while minor bleeding and stent migration were reported in one cohort study (incidence rates 12% and 3%, respectively). Esophageal narrowing was reported exclusively by Chon et al., affecting 20 of 30 patients. Oral intake following VacStent placement was described in six of nine studies: most patients tolerated a liquid diet (Table 4), and in Lange et al., 8/15 progressed to and tolerated a solid diet. Fifteen patients did not achieve clinical success and required additional interventions—ten underwent further EVT with the EsoSponge and five ultimately required surgical management.

**Table 4:** Outcomes and complications of vacstent

Author	Number of patients (*n*)	Complications				Oral intake	Treatment change after VacStent (*n*, patients)
		Sponge dislodgement	Narrowing of the passage	Bleeding	Migration		
Chon et al. [26]	10	3/10	10/10	-	-	Liquids (4/10) None (6/10)	EVT (2) Surgery (1)
Chon et al. [27]	20	-	10/20	-	-	Liquids (10/10)	EVT (7) Surgery (1)
Lange et al. [28]	15	1/15	-	5/41 (12%)	7/41 (3%)	Liquids (13/15) Solid (8/15)	Surgery (2)
Klose et al. [29]	3	-	-	-	-	Liquids (3/3)	-
Pattynama et al. [30]	10	-	-	-	-	Liquids (10/10)	EVT (1)
Marca et al. [31]	1	-	-	-	-	.	-
Mendes et al. [32]	1	-	-	-	-	Liquids (1/1)	-
Shah et al. [33]	1	-	-	-	-	.	-
Ylli et al. [34]	4	-	-	-	-	.	Surgery (1)

* EVT: endovacuum therapy; SEMS* self-expandable metallic stent; (-): none

Long-term follow-up data are very limited: six of nine studies provided no information beyond the treatment period. Three studies reported isolated late strictures only—one case of anastomotic stenosis at 30 days post-treatment (successfully dilated) in Lange et al., one esophageal stricture in Pattynama et al., and anastomotic strictures in La Marca et al. treated with pneumatic dilation. No leak recurrences or other delayed complications were described.

### Causes of heterogeneity among studies

The heterogeneity primarily stemmed from differences in study design. In addition to cohort studies, small case series and case reports were included, as VacStent is a novel intervention and the currently available literature remains limited. Variability was also observed in prior endoscopic interventions used before VacStent placement—such as EVT, SEMS, and endoscopic clips—which introduced heterogeneity in patient characteristics. Furthermore, the VacStent technique itself is not yet standardized. Studies reported different technical details, including variations in negative pressure settings, the volume of saline used, and the intervals for changing the VacStent. Information regarding the defect characteristics, such as leak size, cavity size and exact location, were not available in all studies. The underlying indications for VacStent therapy also differed across studies, contributing to variability in defect characteristics and further complicating data synthesis. Collectively, these sources of heterogeneity precluded the possibility of conducting a meta-analysis, necessitating a predominantly narrative approach to data presentation.

## Discussion

Esophageal perforation and AL are associated with significant mortality, particularly when diagnosis and management are delayed. Current trends are shifting away from surgical repair toward less invasive endoscopic interventions. Among these, the VacStent is a promising endoscopic device combining the benefits of EVT and SEMS, while preserving esophageal patency and allowing oral feeding. Whether VacStent can reduce defect closure time and the length of hospital stay, compared to SEMS or EVT, remains to be established through comparative prospective studies. Based on our findings, VacStent therapy lasted 5 to 14 days, requiring one to three stents for each patient. Technical success was achieved in 100% of cases, without any adverse procedural events. Clinical success was observed in 77%, indicating that some patients required additional interventions. Most patients tolerated liquid diet during treatment, and in one study over 50% progressed to solid diet, highlighting an important benefit of this approach. Furthermore, complications such as significant bleeding or migration were rarely reported, underscoring the VacStent’s favorable safety and efficacy profile. Hence, all of the studies present preliminary data, while the methodological quality was either moderate or poor, warranting cautious interpretation of the findings.

Although the available data are preliminary and derived from small cohort studies across various centers, the technical success rate was 100%, demonstrating feasibility of the procedure in the hands of experienced interventional endoscopists. In two studies, Chon et al. reported an issue with incomplete expansion in the mid-portion of the VacStent following deployment, resulting in narrowing of the esophageal passage. This was attributed to the presence of the sponge, which was thought to counteract the radial expansion force of the stent [26, 27]. However, in the rest of the studies this complication was not observed. Lange et al. later reported that this design limitation was addressed by the manufacturer, and the current version of the VACStent no longer exhibits this narrowing effect [28]. The VacStent is designed with a 14 mm diameter at the central lumen and 30 mm at the flared ends, resulting in a dumbbell shape. This shape enhances anchorage and helps prevent migration– an important benefit compared to conventional SEMS, which have a reported rate of migration rate of up to 36% and often require additional fixation using clips or endoscopic suturing [35–37]. Consequently, a luminal diameter of roughly 14 mm should be anticipated during endoscopic evaluation.

Importantly, all included studies involved mid- or distal esophageal leaks; none evaluated cervical or very proximal defects. The narrower lumen and proximity to the upper esophageal sphincter in the cervical esophagus may render VacStent placement more technically challenging and less well tolerated, limiting device conformability and patient comfort. Consequently, our findings cannot be directly extrapolated to cervical leaks or proximal defects, and dedicated investigations are needed to assess VacStent’s safety, technical feasibility, and tolerability in these anatomically distinct locations.

There was considerable variability among studies regarding the technical parameters of VacStent application, including the suction pressure of the pump, timing of device closure prior to replacement, and the volume of saline instilled to loosen the sponge. According to our findings, pump pressures ranged from 60 to 120 mm Hg. Histological analyses have suggested that effective healing and formation of granulation tissue can occur at pressures as low as 60mmHg [27]. However, higher pump pressures at 120mmHg have been associated with granulation tissue ingrowth into the polyurethane sponge, especially when the VACStent remains in place for more than four days [28, 38]. Notably in some studies, a higher pump pressure to 125mmHg was employed on the first day to ensure firm attachment of the stent to the esophageal wall, followed by a reduction to 75mmHg on the second day to facilitate subsequent removal [30, 32, 33]. In the study by Lange et al., the ease of VacStent was removal was assessed, and it was reported as “easy” in 83% of cases [28]. To aid in removal, it has proven to be effective to discontinue suction at least two hours prior and instill normal saline 0.9% through the drainage catheter [28]. While in most studies saline irrigation was performed immediately before VacStent removal, in some others regular flushing with 20 ml of saline three times a day was applied, to maintain catheter patency and prevent tissue ingrowth [30, 32]. So far, no consensus has been reached on the optimal suction pressure of the pump, the precise time for device closure prior to change and the frequency of saline instillation. These parameters appear to influence the formation of granulation tissue and the ease of VacStent removal.

Given the wide variability in technical parameters among published VacStent series, future studies should adopt a harmonized core protocol. We propose initiating suction at 100 mmHg for the first 12–24 h to optimize early seal formation, then maintaining 60–80 mmHg thereafter to balance wound perfusion with ease of stent retrieval. Saline flushes of 20–40 mL every 8 h will help prevent catheter occlusion, while discontinuing suction at least 2 h before each planned exchange—with a pre-removal bolus of 40 mL of isotonic saline—minimizes tissue ingrowth and facilitates atraumatic device removal. A maximum dwell time of 4 days per device (with earlier exchange if debris accumulates or seal integrity is compromised) balances treatment efficacy against the risk of sponge embedment. Implementing these standardized technical parameters will enhance patient safety and procedural efficiency and will establish a rigorous foundation for future research aimed at elucidating the optimal role of VacStent therapy in the management of esophageal leaks and perforations.

Owing to the retrospective nature of available reports and the lack of prospective, comparative trials, the precise positioning of the VacStent—whether as a first-line therapy or as a salvage/bridging option after other endoscopic measures—remains to be defined. In our pooled cohort, 38% of patients had received at least one prior endoscopic intervention (EVT, SEMS, or clip-based closure) before VacStent placement. To evaluate the potential bias introduced by this “pre-treatment,” we conducted a predefined sensitivity analysis: leak-closure rates were identical at 77% in both treatment-naïve and previously treated subgroups (30/39 vs. 20/26; *p* = 1.00). These findings suggest that earlier endoscopic therapies did not materially influence the observed clinical success of the VacStent. Nonetheless, given the heterogeneity of study designs and patient selection, definitive conclusions regarding optimal sequencing—particularly whether the VacStent should be deployed de novo or reserved for refractory cases—will require randomized, head-to-head comparisons. Several limitations of the VacStent have been identified. Initially, it cannot be used close to the upper esophageal sphincter, as it is associated with significant discomfort for the patient. Another limitation involves the management of large wound cavities. While EVT (Esosponge) can be applied directly inside a cavity, the VacStent remains intraluminal and may be less effective in treating contaminated extraluminal spaces. Additionally, the VacStent has a fixed length of 5 cm, making it mostly applicable for small esophageal defects and necessitates precise placement during endoscopy. In cases involving small cavities, such as early-stage Boerhaave syndrome without pleural collections, the VacStent may serve as a suitable standalone treatment. Additionally, a case report by Shah et al. included in this review, demonstrated the successful use of the VacStent in closing a chronic epithelized fistula, showing that the irrigation of the negative pressure could promote healing even in chronic fistulas [33]. Ultimately, further research is needed to delineate the exact indications for VacStent and to define its role in the management of particular types of esophageal perforations or Als.

One of the key advantages of the VacStent is its allowance for patency of the esophagus, enabling early oral intake. Among the included studies that reported on oral nutrition, the majority of patients tolerated liquid diet. Notably, in the study by Lange et al., eight of fifteen patients were able to consume mashed food without any malfunction of the stent [28]. However, in a study by Chon et al. the administration of liquid nutritional supplements led to malfunction of the VacStent as food particles obstructed the drainage tube [26, 27]. Despite this, overall tolerance of a liquid diet was favorable, which may contribute to improved patient satisfaction and better nutritional status compared to conventional EVT methods such as the EsoSponge. Esosponge is typically placed intraluminally and often obstructs the esophageal passage, precluding oral intake during treatment [30, 39]. While these findings are promising, further studies are needed to evaluate patient-centered outcomes and the nutritional benefits during the VacStent treatment, compared to other methods.

Although no studies have yet directly compared VacStent with other endoscopic techniques, EVT appears to offer several advantages over SEMS. A recent meta-analysis comparing EVT and SEMS for AL following gastric or esophageal oncologic surgery demonstrated that EVT was associated with a higher clinical success rate, fewer required devices, shorter treatment duration, and a lower incidence of short-term complications compared to SEMS [40]. However, another systematic review advised caution in interpreting the apparent superiority of EVT, as most included studies were retrospective in nature, introducing a significant selection bias [41]. Despite the potential benefits of EVT, SEMS retains a key advantage: the ability to maintain luminal patency, thereby allowing the continuation of oral fluid or food intake [42]. As previously discussed, the VacStent aims to integrate the strengths of both approaches—promoting defect closure through negative pressure therapy while preserving luminal continuity—potentially enhancing patient comfort and quality of hospital stay. Unfortunately, there are currently no direct comparative studies evaluating VacStent against EVT or SEMS. Such studies are needed to better define the specific indications, associated risks, and cost-effectiveness of VacStent therapy.

A cost-effectiveness analysis of the VacStent compared to other modalities such as sponge EVT and SEMS, is essential to determine the most efficient approach. In this systematic review an average of 1 to 3 VacStents were required per patient, which could demonstrate increased cost compared to conventional SEMS. Compared to sponge EVT, VacStent has also a higher cost, but it may offer potentially cost savings by reducing the duration of treatment and the number of required endoscopies. The costs of sponge EVT are twice as high compared to those of SEMS treatment due to the higher rate of endoscopies [43–45]. While the initial cost of one VACStent exceeds that of an EsoSponge, the VACStent could possibly reduce treatment duration, hospital stay and amount of EVT-related endoscopies. Furthermore, VacStent may offer additional advantages in terms of nutritional intake, which could contribute to faster recovery [28, 30]. However, none of the studies included in this systematic review conducted a cost analysis, and a formal economic evaluation of VacStent therapy is currently lacking. Future prospective studies should systematically capture device and comparator costs, hospital and ICU stay durations, procedural and complication expenses, and patient quality-of-life outcomes. Decision-analytic models developed with health economics expertise will be essential to assess VacStent’s incremental cost-effectiveness.

To date, no randomized or controlled trials have directly compared the VacStent with standard EVT or SEMS. Existing VacStent research comprises only single-arm studies assessing safety, technical performance, and clinical outcomes. Two ongoing prospective trials—DRKS00016048 in Germany and NCT04884334—are collecting real-world data on VacStent use in esophageal, upper GI, and colorectal leaks, but lack EVT-only or SEMS-only comparator arms. Ideally, a multicenter, randomized controlled trial should be conducted, randomizing patients with comparable esophageal leaks in a 1:1 ratio to receive either the VacStent or standard EVT (or SEMS). Such a trial should stratify participants by leak size and etiology, employ blinded adjudication of endoscopic closure, and be powered to demonstrate non-inferiority or superiority in primary leak-closure rates. Secondary endpoints ought to include time to healing, number of interventions, adverse events, patient comfort scores, and cost-effectiveness, all analyzed on an intention-to-treat basis.

This systematic review has several limitations. The data extracted from the included studies were preliminary, often involving initial cohorts of consecutive patients and reflecting early experiences across different centers. Additionally, the overall quality of evidence was limited by study design, since three of the included studies were case reports and two were case series. According to the quality assessment of the included studies, the methodological quality was generally moderate, indicating a lack of rigorous study design. Also, the significant heterogeneity in reported outcomes and study protocols prevented the performance of a meta-analysis and the findings were presented in a narrative manner. Moreover, we restricted our inclusion to full-text, English-language publications to ensure data quality and methodological transparency, and excluded conference abstracts due to their typically limited detail. We acknowledge that these criteria may have introduced publication bias by omitting relevant non-English studies and grey-literature reports. Given the limited number of studies (*n* = 9) and their heterogeneity—and the absence of quantitative meta-analysis—formal statistical assessments of publication bias (e.g., funnel plots or Egger’s test) were not feasible. Future reviews incorporating multilingual searches and grey literature could help provide a more comprehensive estimate of VacStent efficacy. Despite these limitations, this review represents the first systematic synthesis of available evidence for the use of the VacStent. It provides an overview of the current clinical experience and highlights the potential of this novel endoscopic technique, while also emphasizing the need for more prospective and comparative studies in this area.

## Conclusions

The VacStent appears as a promising tool in the armamentarium of endoscopists and surgeons for managing esophageal perforation and AL. By combining the benefits of EVT and SEMS, while allowing oral intake, it could be established as an optimal endoscopic intervention. However, its precise role within the current treatment landscape needs to be defined. More evidence through prospective comparative studies will delineate the advantages of the VacStent in comparison to other established endoscopic techniques.

## Data Availability

No datasets were generated or analysed during the current study.

## Abbreviations

GEJ: gastroesophageal junction
AL: anastomotic leak
GI: gastrointestinal
SEMS: self-expanding metallic stent
EVT: endoscopic vacuum therapy
PRISMA: Preferred Reporting Items for Systematic Reviews and Meta-Analyses
MeSH: Medical Subject Headings
JBI: Joanna Briggs Institute
MINORS: Methodological Index for Non-Randomized Studies
